# A Two-Year Follow-Up Assessment of Decreasing Crestal Bone Levels Around Dental Implants in Patients Rehabilitated With Mandibular Implant Overdentures

**DOI:** 10.7759/cureus.29044

**Published:** 2022-09-11

**Authors:** Prince Kumar, Brajesh Dammani, Monica Jaideep Mahajani, Vinay H Vadvadgi, Rashmi Jawade, Mohit V Patil

**Affiliations:** 1 Department of Prosthodontics, Rama Dental College Hospital and Research Centre, Kanpur, IND; 2 Department of Prosthodontics, Vidarbha Youth Welfare Society's (VYWS) Dental College and Hospital, Amravati, IND; 3 Department of Periodontology, Dr Hegdewar Smruti Rugna Seva Mandals (HSRSM) Dental College and Hospital, Hingoli, IND; 4 Department of Periodontology, Rural Dental College, Pravara Institute of Medical Sciences, Loni, IND; 5 Department of Periodontology, Dr Rajesh Ramdasji Kambe Dental College and Hospital, Akola, IND; 6 Department of Prosthodontics, SMBT Dental College and Hospital, Sangamner, IND

**Keywords:** osseointegration, overdenture, implant, bone loss, cone beam computed tomography

## Abstract

Aim: This two-year follow-up study was aimed to evaluate declining crestal bone levels around dental implants in patients rehabilitated with mandibular implant-supported overdentures. A three-dimensional advanced radiographic tool, cone beam computed tomography (CBCT), was utilized as radiographic aid in this study.

Materials & Methods: A total of 15 patients wearing mandibular implants supported overdentures were studied for two years. Randomization and strict inclusion/exclusion criteria were followed during study execution. Complete dentures were fabricated with standard methods, which were later anchored by a bilateral implant in the mandibular jaw. Bone loss at all four surfaces in all studied implants was estimated by the cone beam computed tomography (CBCT) technique. These assessments were done at postoperative follow-up periods of six, 12, 18, and 24 months. Duly signed and informed consent was obtained from all participating patients.

Statistical Analysis and Results: The statistical analysis was completed by the software IBM Corp. Released 2013. IBM SPSS Statistics for Windows, Version 22.0. Armonk, NY: IBM Corp. All relevant data was entered into it to be analyzed with suitable statistical tests. Out of all 15 studied patients, 11 were male, and four were female. P-value was very significant for the age range 35-40 years (0.01). In all instances, the lingual surface showed minimum, while the distal surface showed maximum bone loss when seen at all postoperative phases. Grossly, the mean bone loss ranged between 0.14-0.45. P-value was highly significant for the measurements made at the lingual and distal sides of implants (for both B and D positions). A comparison of both study groups by one-way ANOVA confirmed a highly significant p-value for estimations done between the groups (0.001).

Conclusion: Within the limitations of the study, the authors confirmed that crestal bone levels showed a clear decreasing pattern in the postoperative phases. Since these deleterious processes can compromise long-term prosthesis success, operators should consider all these facts while planning to implant an overdenture prosthesis in the lower jaw.

## Introduction

Crestal bone loss is a common clinical dilemma that affects the long-term clinical success of implant prostheses. This clinical problem not only affects longevity but also jeopardizes the oral environment to infectious attacks [1-3]. Repair related to alveolus is a continuous process that is greatly inhibited by microbial attacks. These infections can be acquired from outside or nearby anatomical structures. Since the mouth is considered the first door of microbial ingress, oral structures are highly prone to get these infections. In addition, prostheses like complete dentures and fixed partial dentures are also highly susceptible to bacterial attack [4,5]. Dental implants are also at risk of getting infections from the oral cavity. Mostly implant-related issues are arising from the surrounding alveolar bone and other mechanical factors. If the nearby alveolar bone is compromised or infected, it tends to resorb at a higher rate [6,7]. The resorption of the bone eventually leads to the failure of osseointegration and the failure of implants. In the literature, many clinicians have tried several clinical and methodological ways to minimize these clinical dilemmas [8-10]. However, none of them has completely succeeded in preventing bone loss surrounding dental implants. Many of them strongly believe that alveolar bone loss is one of the prime factors in implant failure. These bony activates are frequently associated with other mechanical factors like occlusal force and occlusal scheme [11,12]. Implant overdentures are much liked by completely edentulous patients because of the poor retention and stability of the traditional lower denture. Implant retained overdenture offer excellent retention and stability to the denture, somewhat like a fixed prosthesis. In addition, researchers had already warned about the failures of implants supported overdenture in different circumstances [13,14]. It is, therefore, very imperative to have all the measures required for successful implants, like strict sterilization, an ideal occlusal scheme, balanced forces, and optimal oral hygiene. So, considering all these facts, this two-year follow-up study aimed to evaluate declining crestal bone levels around dental implants in patients rehabilitated with mandibular implant overdentures. Three-dimensional advanced cone beam computed tomography (CBCT) was utilized as radiographic aid in this study.

## Materials and methods

This study was planned and executed in the department of prosthodontics of the institute. The outline of the study was prepared and presented to the institutional ethical committee for approval. This study proposal was also forwarded to the institutional scientific committee for quality and worthiness check. Following these approvals (Ethical Clearance No. 20/IEC/RDCHRC/2022/Cmltv298), the study started, which mainly included completely edentulous patients restored with mandibular implant-supported overdentures in the last two years.

A total of 15 patients were included in which mandibular implant overdentures have been fabricated in the department. Out of these, 11 were males, and four were female patients. The study model was a prospective cohort wherein all these patients were selected by a simple random sampling procedure. A simple random sampling procedure ensured balancing among the participants during the study period. Inclusion criteria included a) patients in the age range of 30 to 55 years, b) patients reported and treated in the institution, and c) patients with completely erupted permanent maxillary dentition. Exclusion criteria included a) patients with a known history of loss of follow-up, b) patients already having some bone graft therapy or medication, c) high-risk patients with known episodes of osteoporosis, and d) nonresponsive patients even after repeated contacts and trials. Other local factors like smoking, unusual jaw relations, and uncooperativeness are also considered and not included. Bilateral osseointegrated implants were placed at B and D positions in all 15 patients interested in superior retention and stability of the lower denture.

The clinical procedure followed all standard and mandatory steps like a pilot drill, usage of a surgical template, and paralleling pins. All radio-graphical planning and bony assessments were attempted by cone beam computed tomography. After placement of implants, patients were asked to wait for three months to ensure complete osseointegration between alveolus and implants. Standard complete (over) dentures were fabricated after a competition of three months. Standard ball abutments were placed in the usual manner, and corresponding metal housing was placed in the lower denture at corresponding locations. For studying the bone levels or bone losses, we have divided the implants into two groups. Bone losses were estimated by the CBCT software by comparing them with the previous radiographic data of similar locations. Group 1 consists of all implants placed at B positions, and Group 2 consists of all implants placed at D positions. Since study subjects were similar in both groups, randomization was not needed accordingly. During the post-insertion follow-up period, bone levels were checked at different timings, i.e., six, 12, 18, and 24 months. These radiographic evaluations were attempted at the mesial, distal, buccal, and lingual sides of both implants in all patients. All participating patients were informed in detail about the study, including benefits, risks, alternatives, compensations, emergency contacts, and freedom to participate. Written and signed informed consent were obtained accordingly. Data and inferences were compiled in a table and sent for necessary statistical analysis. A p-value less than 0.05 was taken as significant (p< 0.05). 

The data entry was completed into spreadsheets and sent for analysis. Variables were recorded as per software requirements (clear, concise, consistent, no copy). IBM Corp. Released 2013. IBM SPSS Statistics for Windows, Version 22.0. Armonk, NY: IBM Corp. was used for details statistical analysis of the data. Post-hoc analysis was avoided. Post-hoc analysis was avoided since it can lead to erroneous estimates and results.

## Results

Table 1 shows that all participants were in the age range of 30 to 55 years, with 11 male and four female patients. The age group of 35-40 years has a maximum of six patients. P-values were highly significant here (0.01). A minimum of one patient was identified in the age group of 51-55 years. P-value was not significant here.

**Table 1:** Age- & gender-based assessment of participating patients

Age Group (Yrs)	Male	Female	Total	P value
30-34	2	1	3	0.08
35-40	5	1	6	0.01
41-45	2	1	3	0.50
46-50	1	1	2	0.10
51-55	1	0	1	0.20

Table 2 illustrates the necessary statistical explanation for Group 1, in which 15 implants were placed at position B.

**Table 2:** Essential statistical explanation for Group 1 (n = 15, position ‘B’) *: statistically significant L: Lingual, B: Buccal, M: Mesial, D: Distal, CI: Confidence interval

Side		Mean Bone Loss (in Months)				Mean	Std. Deviation	Standard Error	95% CI	Chi-Square Value	df	p value
		6 Month	12 Month	18 Month	24 Month							
L		0.14	0.16	0.19	0.22	0.17	0.837	0.430	1.23	2.435	1.0	0.01*
B		0.22	0.27	0.32	0.36	0.29	0.203	0.938	1.96	2.303	2.0	0.20
M		0.23	0.29	0.36	0.42	0.32	1.531	0.857	1.76	2.209	1.0	0.10
D		0.39	0.40	0.41	0.45	0.41	0.803	0.102	1.90	1.264	1.0	0.02*

These bone loss assessments were attempted at all four-intended postoperative phases. There was no clinical evidence of attrition in all four postoperative phases. On average, the lingual surface showed minimum, while the distal surface showed maximum bone loss when checked at all phases.

At the lingual surface, the mean bone loss ranged between 0.14-0.22; at the buccal surface, it ranged between 0.22-0.36; at the mesial surface, it ranged between 0.23-0.42; at the distal surface, it ranged between 0.39-0.45. P-value was highly significant for assessments made at lingual and distal surfaces.

Table 3 demonstrates the essential statistical explanation for Group 1, in which 15 implants were placed at position D. Generally, the lingual surface showed minimum, while the distal surface showed maximum bone loss when estimated at all postoperative phases. At the lingual surface, the mean bone loss ranged between 0.16-0.23; at the buccal surface, it ranged between 0.24-0.33; at the mesial surface, it ranged between 0.25-0.38; at the distal surface, it ranged between 0.40-0.44. P-value was highly significant for assessments made at lingual and distal surfaces, and this stands true for both the studied locations (B & D).

**Table 3:** Essential statistical explanation for group 2 (n = 15, Position ‘D’) *: statistically significant L: Lingual, B: Buccal, M: Mesial, D: Distal, CI: Confidence interval

Side	Mean Bone Loss (in Months)					Mean	Std. Deviation	Standard Error	95% CI	Pearson Chi-Square Value	df	p value
	6 Month		12 Month	18 Month	24 Month							
L	0.16		0.17	0.19	0.23	0.18	0.938	0.389	1.69	1.032	1.0	0.01*
B	0.24		0.26	0.29	0.33	0.28	0.032	0.930	1.92	1.827	2.0	0.60
M	0.25		0.28	0.32	0.38	0.30	0.637	0.536	1.02	2.038	1.0	0.50
D	0.40		0.41	0.43	0.44	0.42	0.854	0.712	1.45	1.029	1.0	0.01*

Table 4 shows a comparison of both study groups by one-way ANOVA. P-value was very significant for assessments done between groups (0.001).

**Table 4:** Assessment among the two study groups by one-way ANOVA [for group 1, 2] *: statistically significant

Variables	Degree of Freedom	Sum of Squares ∑	Mean Sum of Squares m∑	F	p value
Between Groups	2	2.746	1.029	2.3	0.001*
Within Groups	24	4.520	0.335	-	
Cumulative	137.03	8.039	-		

## Discussion

Bone loss associated with dental implants is one of the most studied and experimented with topics in implant dentistry. It has been widely debated and researched across the globe for decades still one has formulated a concrete statement or concept [15-17]. Recently many pioneer researchers, including Young et al., studied variants of CBCT and its role in dental implantology, and they claimed that CBCT is an ideal radiographic tool for estimating alveolar bone losses [18]. Umetsubo and associates also validated the perception and accuracy of CBCT in planning implants in the lower jaw [19]. Peterson and colleagues also studied alveolar bone and its relation to implants, dehiscence, and fenestration. They concluded that only advanced radiographic aid is capable of locating minute details in the jaw bones [20]. Al-Saleh and Januário et al. also highlighted the importance of bone loss and related implant failure. They also emphasized the relationship between implant failure and aesthetics. The study results were highly predictable and comparable with ours [21-22]. Pette reported on incidental findings of CBCT imaging and their clinical significance [23]. Tward and Kabashima et al. highlighted the significance of three-dimensional imaging in detecting any soft tissue dilemmas. Even in our study, we noticed the similar significance of CBCT imaging [24,25].

Nickenig and colleagues studied in detail marginal bone loss in differently machined implants with dissimilar surface treatments. They concluded that different designs and surface treatments greatly affect the surrounding bone loss and associated success. Their study findings were in accordance with ours principally in terms of bone loss with one particular design [26]. Shokouhi and others, in 2022, studied the effect of radiotherapy on the long-term success of dental implants and states that the magnitude and duration of radiotherapy in the implant region significantly affect implant longevity. We also agreed with their findings and followed their recommendations during our study [27]. Mortazavi et al., in 2021, studied factors of bone loss in the Korean population. They also emphasized the importance of strict sterilization during osteotomy and optimal oral hygiene in follow-up periods. Even in our study, we tried our best to follow these sterilization guidelines to minimize bone loss [28]. Wilson has also experimented to resolve the confusion between metallosis and implant bone loss. Their study was of great importance since it covered all four portions of periodontium [29].

The limitation of this study is that the authors included only lower arch and studied fixed implant locations in terms of mandibular implant overdenture. The authors did not emphasize the effects of force exertion by existing maxillary natural dentition. Moreover, we studied a smaller sample size due to institutional limitations of patient flow. Therefore, we recommend other studies with a larger sample size, which could fairly establish concrete guidelines for these prospects. 

## Conclusions

Within the limitations of the study, the authors stated that crestal bone loss is a clinical phenomenon that is clearly evident on all surfaces of the osseointegrated implant. This also stands true in cases of mandibular implant overdentures. In our study, bone levels showed a clear decreasing pattern in the postoperative phases up to the first two years. Both the studied locations (B & D) illustrated somewhat similar bone loss patterns with minimum values on the lingual side and maximum values on the distal sides. Therefore, clinicians must be aware of such phenomenon while estimating the long-term prognosis of implant overdentures in mandibular arches. The authors also emphasized the precisions of data made by CBCT since it revealed even minute details or measurements which is otherwise unrecognizable by routine radio-graphical methods. 
